# Supplementary material for: Suppressing torsional buckling in auxetic meta-shells
Source: Nat Commun. 2024 Aug 14;15:6999. doi: 10.1038/s41467-024-51104-3 (PMC11324657; doi:10.1038/s41467-024-51104-3)
Supplement: Supplementary file 1 — Supplementary Information [file 41467_2024_51104_MOESM1_ESM.pdf]

# Supplementary Information: Suppressing torsional buckling in auxetic meta-shells

Aref Ghorbani*[1], Mohammad J. Mirzaali[2], Tobias Roebroek[1], Corentin Coulais[3], Daniel Bonn[3], Erik van der Linden[1], and Mehdi Habibi†[1]

[1]Laboratory of Physics and Physical Chemistry of Foods, Wageningen University, 6708 WG Wageningen, The Netherlands
[2]Department of Biomechanical Engineering, Delft University of Technology, 2628 CD Delft, The Netherlands
[3]Institute of Physics, University of Amsterdam, 1098 XH Amsterdam, The Netherlands

July 27, 2024

*Corresponding author. Email: aref.ghorbani@wur.nl
†Corresponding author. Email: mehdi.habibi@wur.nl

# Supplementary Information

## Critical shell thickness

If the meta-shell is too thin, the structural design and corresponding properties become irrelevant, as out-of-plane buckling will be immediately triggered. All meta-shells discussed in the main text exceed this limit, with a shell thickness of $T = R_{\text{max}} - R_{\text{min}} = 5$mm. We determine the critical thickness of the meta-shell with $\theta = \pi/6$ (see **Supplementary Figure** 1a), above which auxeticity dominates, through finite element simulations. In **Figure** b, we illustrate torque as a function of torsional angle for meta-shells with shell thicknesses ranging from $T = 1$mm to 5mm, in 0.5mm intervals. The obtained maximum torque, $\tau_{\text{max}}$, and twist angle, $\varphi_{\text{max}}$, before buckling indicate that the mechanism is valid above the critical shell thickness of $T > 4$mm, and torsional buckling is triggered below this limit (**Figures** 1c and d).

## Buckling under compression

Here, we observe a transition in the buckling mode depending on the orthotropy orientation. In **Supplementary Figure** 2a, we compare the buckling modes of the meta-shells with different unit-cell orientations. We observe that the meta-shells with zero or small unit-cell rotation (e.g., $\theta = 0$ and $\pi/12$) show a side buckling, like the Euler buckling of a beam. On the contrary, the meta-shells with $\theta = \pi/6$ and $\pi/4$ only buckle internally, keeping the shell's cylindrical morphology intact under large compression strains.

In **Supplementary Figure** 2b, we show the rescaled compression stress as a function of the applied compression strain. We observe that the effective Young's modulus of the meta-shells in the pre-buckling regime, $\delta < 0.02$, decreases as a function of their unit-cell orientation, $\theta$ (**Figure** 2c), representing a softening by increasing $\theta$. Although the buckling mode depends on the unit-cell orientation, the critical compression stress for buckling transition is almost constant ($\sigma_c \approx 0.006Y$) for all meta-shells (**Supplementary Figure** 2d). But the compression strain that corresponds to the onset of buckling , $\delta_c$, is rather higher for the meta-shells with $\theta = \pi/6$ and $\pi/4$, where the buckling occurs internally, and the meta-shells softens (**Supplementary Figure** 2e). However, the maximum stresses and strains that the meta-shells with $\theta = \pi/6$ and $\pi/4$ can resist with keeping their cylindrical morphology are an order of magnitude higher than their value at the onset of local buckling, shown by open circles in **Supplementary Figure** 2d and e, that indicates the high compaction capacity and compressibility ($\sim 50\%$) of these meta-shells.

## Shear modulus of a tilted beam

We can predict the shear modulus of the rotated unit-cell using a simple Hookean spring with the length of $a_0$, initially tilted by $\theta$ with respect to the vertical direction (**Supplementary Figure** 3). The shear deformation is applied in the horizontal direction and the force is given by $F_s = k\delta l \sin(\theta - \delta\theta)$, where $\delta l$ is the applied strain, $k$ is the spring constant, and $\delta\theta$ is the change in the angle. Using the geometrical constraints and the Taylor series, we can obtain the equilibrium equation,

$$F_s = ka_0 \cos\theta \sin^2\theta\gamma, \tag{1}$$

where $\gamma = \delta x/h_0$. If the effective shearing area is $A$, the shear modulus is given by $G_s = (ka_0/A)\cos\theta\sin^2\theta \approx (ka_0/A)\theta^2$, which is consistent with our observation of the quadratic relationship between the shear modulus and unit-cell orientation.

## Local stiffness calculation

The stiffness modulus is given by $g_s = (h/J)(d\tau/d\varphi)$, where $d\tau/d\varphi$ is the differential of the torque with respect to torsion, and calculated numerically from the experimental data. Very small $d\varphi$ steps may produce large fluctuations in local stiffness due to experimental inaccuracy in the torque response. Therefore, to mitigate the fluctuations we increased the step size of the torsional deflection to $d\varphi = 110$mrad and kept enough data points. These fluctuations are not present in the finite element results.

## Energy perspective

We observe considerable differences in the energy cost to reach the buckling threshold in both torsion and compression experiments (**Supplementary Figure** 4). The maximum energy stored in the helical meta-shells under torsion is significantly higher. This observation confirms that the stability landscape is significantly tunable via designing orthotropy orientation in meta-shells.

## Uniaxial experiments on the bulk samples

In our analysis of the elastic behaviors of the composition, we examine the response of bulk samples. We investigate a bulk shell with the identical dimensions as the meta-shells and a bulk cube with the width of 2mm, under uniaxial loading. The bulk shell before deformation and after buckling is depicted in **Figure** 5a, and the stress-strain response of both samples are illustrated in **Figure** 5b.

Additionally, we investigate the tensile properties of the 3D printed composition through a tensile test conducted on a dogbone sample with the thickness of 4mm, total length of 120mm, and with of 8mm at the thin section. The response of the system is visually demonstrated in **Figure** 5c, and the stress-strain curve upon tensile is shown in **Figure** 5d.

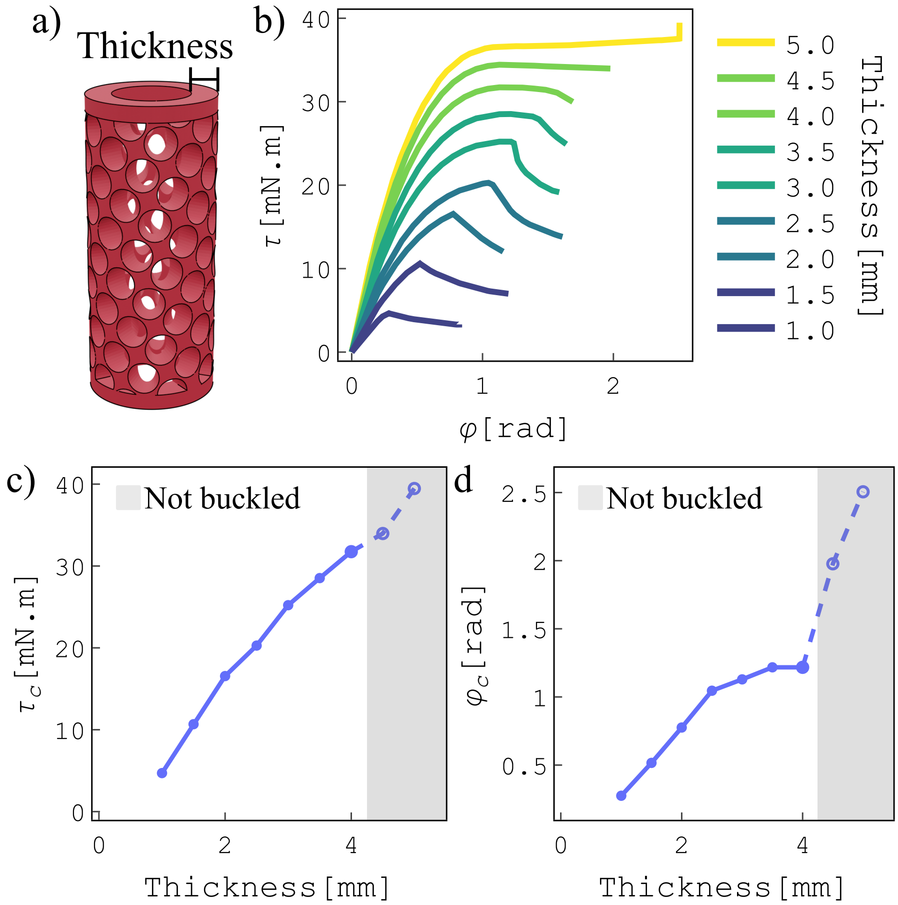


Supplementary Figure 1: Critical shell thickness: a) Visualization of the meta-shell thickness. b) Torque response plotted against the torsional angle for meta-shells with various thicknesses obtained from the FE analysis. c) and d) Maximum torques (c) and torsional angles (d) obtained before buckling is triggered, plotted as functions of the shell thickness.

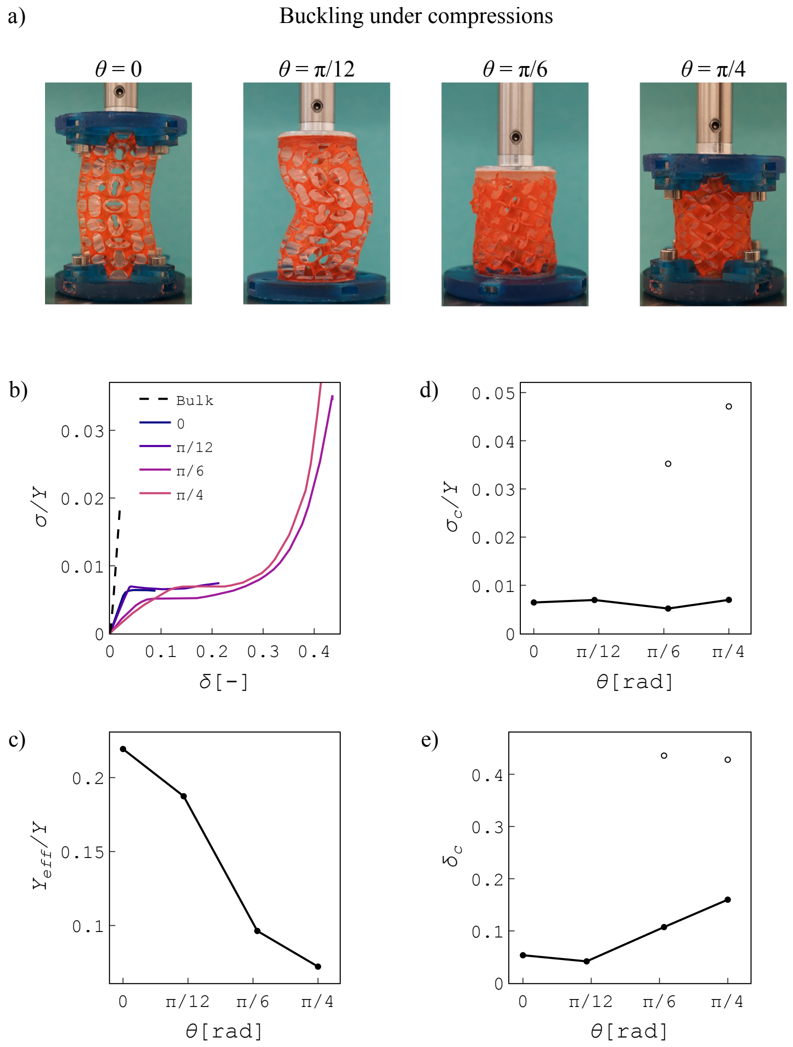


Supplementary Figure 2: The meta-shells under compression: a) The meta-shells with $\theta = 0$ and $\theta = \pi/12$ show global buckling (side buckling) under compression, while the meta-shells with $\theta = \pi/6$ and $\theta = \pi/4$ buckle internally, keeping the meta-shells intact. b) Rescaled compression stress as a function of compression strain, and (c) rescaled effective Young's modulus of the meta-shells as a function of the unit-cells orientation, $\theta$. The onsets of buckling under compression, determined by rescaled stress (d) and compression strain (e), are shown as a function of $\theta$. The empty circles in d and e show the maximum values experimentally obtained, where meta-shells remain straight, and side buckling does not occur yet.

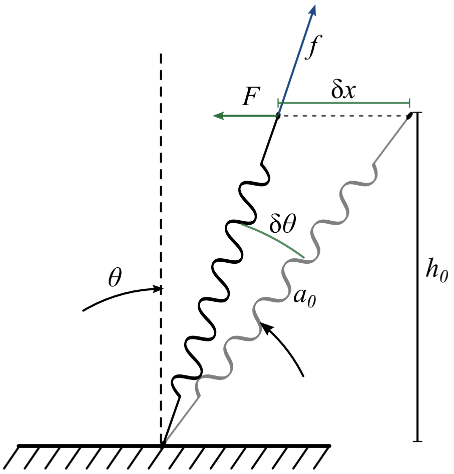


Supplementary Figure 3: A tilted Hookean spring sheared by $\delta x$.

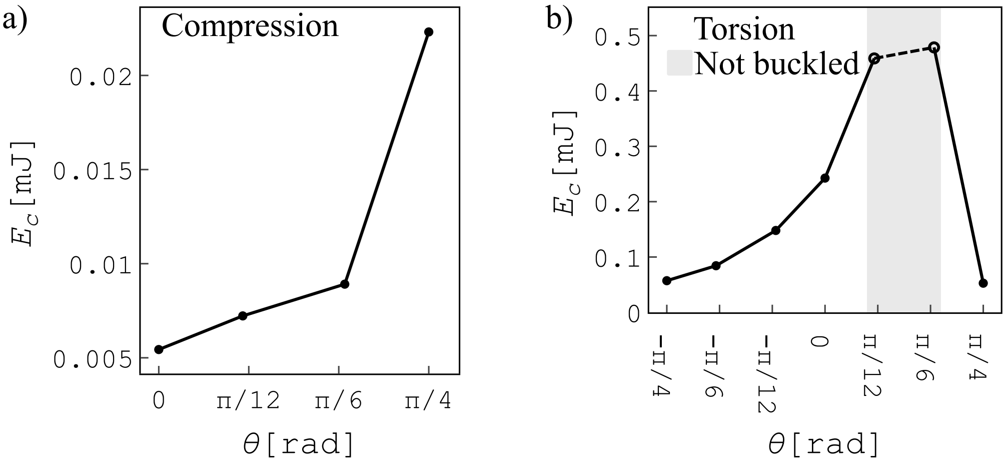


Supplementary Figure 4: Critical buckling energy is stored in the meta-shells under compression (a) and torsion (b) before any buckling occurs. No torsional buckling is observed in the gray area, but we display the maximum energy stored upon the maximum torque limit of the equipment, displayed by open circles.

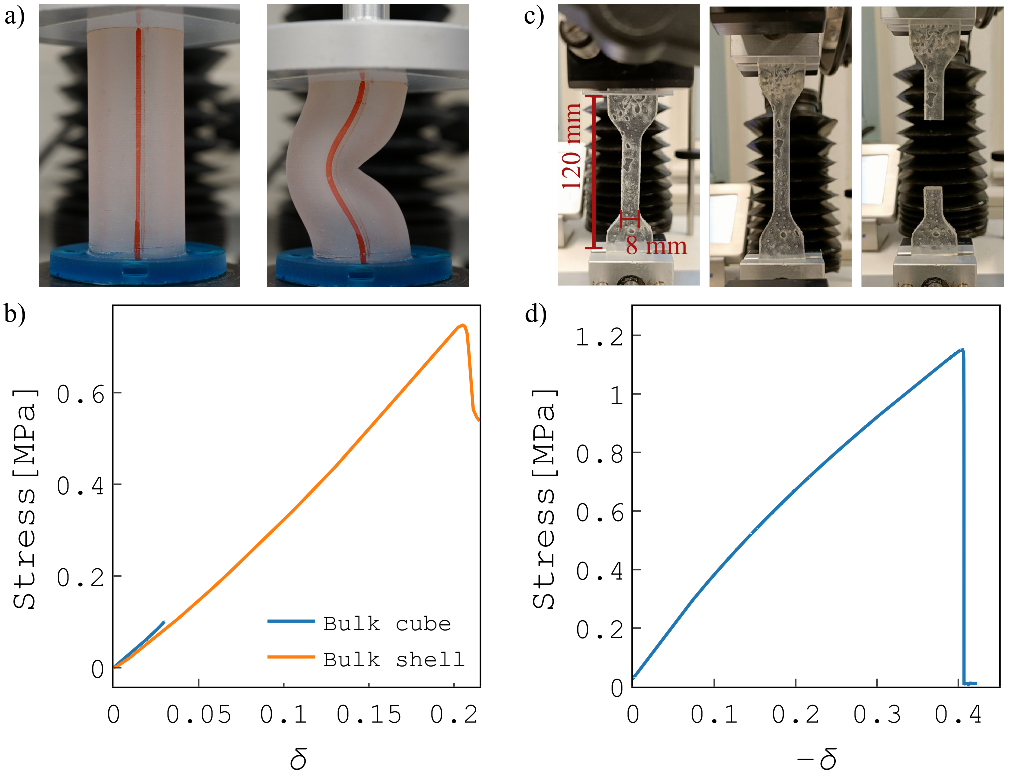


Supplementary Figure 5: Experiments on the bulk samples: a) a) A bulk shell with identical dimensions to the meta-shells (left) showing buckling under uniaxial compression (right). b) The stress-strain curve of the bulk shell and bulk cube with a width of 2 mm during uniaxial compression. c) The dogbone sample, 3D printed with the same materials (left), illustrated when stretched (middle) and ruptured (right) under the tensile test. d) The stress-strain curve resulting from the tensile experiment conducted on the dogbone sample.
