# Supplementary material for: Suppressing torsional buckling in auxetic meta-shells
Source: Nat Commun. 2024 Aug 14;15:6999. doi: 10.1038/s41467-024-51104-3 (PMC11324657; doi:10.1038/s41467-024-51104-3)
Supplement: Supplementary file 3 — Description of Additional Supplementary Files [file 41467_2024_51104_MOESM3_ESM.pdf]

Description of Additional Supplementary Files

File Name: Supplementary Video 1
Description: **Buckling and stability of meta-shells under large torsional deflection.** Response of meta-shells with unit-cell rotation angles of $\theta=0$, $\pi/6$, and $\pi/4$ to clockwise torsion. The helical meta-shell with unit-cell rotation angles of $\theta=\pi/6$ uniformly shrinks and avoids buckling when twisted.

File Name: Supplementary Video 2
Description: **Local contraction of a twisted shell with opposite chirality at the top and bottom halves.** Twisting of a shell made of two helical meta-shells with opposite unit-cell rotation angles of $\theta=+\pi/6$ and $-\pi/6$ leads to its local contraction in the top half when twisted in the clockwise direction and bottom half when twisted in the counterclockwise direction.

File Name: Supplementary Video 3
Description: **Pumping and pulsatile flow using torsion-induced contraction mechanism.** Pumping via twisting using a helical meta-shell with $\theta=\pi/6$ and creating a pulsatile flow upon a twist-release mechanism.
